# Characterization and dynamics of the soluble immunological microenvironment in melanoma patients undergoing radiotherapy

**DOI:** 10.1186/s13014-022-02167-3

**Published:** 2022-11-28

**Authors:** Michael Oertel, Katrin Borrmann, Andrea Baehr, Hans Theodor Eich, Burkhard Greve

**Affiliations:** 1 Department of Radiation Oncology, University Hospital Muenster, Albert-Schweitzer-Campus 1, Building A1, 48149 Muenster, Germany; 2 Department of Radiotherapy and Radiation Oncology, University Medical Center Hamburg-Eppendorf, Hamburg, Germany

## Abstract

**Background and purpose:**

Malignant melanoma constitutes an aggressive tumor of the skin, the pathogenesis of which is influenced by immunological processes. In this context, the influence of radiotherapy (RT) on inflammatory markers has not been studied in detail, yet.

**Materials and methods:**

In this prospective analysis, 28 patients were recruited, 24 of these could be included for further analysis. According to protocol, patients underwent three blood-draws: before, after half of RT-fractions and after completion of RT. Serum levels of programmed death-ligand (PD-L) 1 and 2, interleukin 6 and cytotoxic t-lymphocyte-associated protein 4 were assessed via enzyme-linked immunosorbent assay and compared to healthy volunteers. Correlation with clinical data was attempted.

**Results:**

Comparing patients with healthy volunteers, a significant difference in the mean baseline serum-level of PD-L1 (90.1 pg/ml vs. 76.7 pg/ml for patients vs. control, respectively; *p* = 0.024) and PD-L2 (4.4 ng/ml vs. 8.7 ng/ml; *p* = 0.04) could be found. Increased levels of PD-L1 were only found in patients with prior immunotherapy. There was a tendency for higher interleukin 6 levels in the patients (8.5 pg/ml vs. 0.6 pg/ml; *p* = 0.052). No significant differences in serum levels could be found between the three time points.

**Conclusion:**

The present study reveals a characteristic immunological pattern for melanoma patients in comparison to healthy controls. Future studies will have to focus on a putative correlation between immunological markers and clinical outcome parameters.

## Highlights

- Malignant melanoma patients reveal a characteristic and distinct immunological pattern.
- Elevated levels of soluble PD-L1 induced by previous immunotherapy were found.
- There were decreased levels of soluble PD-L2 in comparison to healthy controls.
- Radiotherapy did not result in significant dynamics of the soluble parameters in the (short-time) course of disease.

## Introduction

Malignant melanoma (MM) is an aggressive skin tumor with rising incidences, the pathophysiology of which is mediated by immunological processes [1–5]. Ipilimumab, an antibody against the cytotoxic t-lymphocyte-associated protein 4 (CTLA-4), a cardinal regulator in the tumor inflammatory micro-milieu, successfully increased overall survival in metastatic patients [4, 5]. Another immunomodulatory protein, programmed-death ligand 1 (PD-L1), is also targeted by a specific antibody [6]. PD-L2 constitutes a further ligand for the PD-receptor [7] which is not addressed by therapeutic antibodies, yet. Likewise, interleukin 6 (IL-6), as an inflammatory mediator, has a complex role in tumor regulation with attributed pro- and anti-neoplastic properties [8].

Recent studies have demonstrated the presence of soluble PD-L1 (sPD-L1) both in vitro and in vivo, which may be measured via an enzyme-linked immunosorbent assay (ELISA) and which has putative prognostic value [9–14].

Soluble markers are advantageous as they are accessible via blood draw and may be used in diagnosis and therapeutic monitoring (liquid biopsy). However, studies on this technique in MM are sparse and often lack patients undergoing RT or detailed information on the dynamic of the parameters. Therefore, the aim of the current analysis was to investigate four important inflammatory parameters (sPD-L1, sPD-L2, IL-6, CTLA-4) in MM patients treated by RT in order to gain insights into their immunological role.

## Material and methods

### Ethical approval and patient data

The study was designed as a prospective biomarker protocol and approved by our institutional review board (2016-664-f-S with an amendment on 02nd March 2021 for the analysis of PD-L2, CTLA-4 and IL-6) and executed according to the principles of the declaration of Helsinki. Samples from 20 healthy volunteers were received from the institute of transfusion medicine and served as a control group. Written informed consent was given by all patients and volunteers involved.

Patient data on demographics, radiotherapy treatment, toxicities, laboratory values and outcome were collected from the clinical files and the hospital’s information system (Orbis, Dedalus Health Care, Bonn, Germany), which provided toxicity documentation, doctors’ letters and imaging. Follow-up was recorded till April 2022.

### Study flow

Blood samples were taken before the beginning of RT (pre-RT), after completion of half of the RT-series (half fractions; mid-RT) and immediately after the completion of RT (post-RT). After collection of 7.5 ml venous blood by sterile venipuncture using a monovette with clotting activator (S-Monovette, Sarstedt, Nümbrecht, Germany), blood samples were stored at room temperature for 30 min to ensure coagulation. Monovettes were then centrifuged for 10 min at 1500×*g* at room temperature and the supernatant (serum) was transferred and aliquoted into 2 ml cryotubes (Diagonal, Münster, Germany). Serum samples were immediately stored in a freezer at -80 °C until further analysis.

The quantification of the serum parameters PD-L1, PD-L2, CTLA-4 and IL-6 was done using ELISA kits (Bio-Techne, Wiesbaden, Germany) according to the manufacturer’s protocols adapted concerning the optimal serum dilution.

### Statistical analysis

Toxicities were graded according to common terminology criteria for adverse events version 5 [15]. Response to treatment was assessed via radiological follow-up.

Statistical analyses were done with SPSS version 28 (IBM, Armonk, New York, USA). Post-hoc power analyses were performed with G*Power version 3.1 (Heinrich-Heine University, Duesseldorf). Progression-free survival (PFS), local control and overall survival (OS) were calculated starting from the first day of RT to the respective event using the Kaplan–Meier method and the log-rank test to compare various factors. Comparisons between the blood level of healthy participants and patients or between the different blood draw dates were done with a two-sample or one-sample t-test, respectively. Categorical variables were compared by means of a two-sided exact Fisher test. A *p* value < 0.05 was considered as significant.

## Results

Overall, 28 patients were recruited to this prospective study with 4 patients being excluded from further analysis (unavailability/poor quality of blood samples, patients not undergoing RT). Details on the study collective are given in Table 1.

**Table 1:** Patient and treatment characteristics

	n/Median (Range)
Number of patients	24
Age at initial diagnosis	63.7 y (27.9 y -78.4 y)
Age at RT	67.4 y (29.3 y – 79.3 y)
Initial Breslow level	3 (1.1–15)
Initial clark stage	
3	1
4	9
Unknown	14
Stage at initial diagnosis	
1B	2
2A	2
2B	1
2C	4
3B	6
3C	2
4	4
Unknown	3
Stage at RT	
3B	1
3C	2
4	19
Unknown	2
Prior immunotherapy	11
Interferon	4
Nivolumab plus ipilimumab	4
Nivolumab	3
Concomitant immunotherapy	8
Nivolumab	5
Pembrolizumab	2
Nivolumab plus ipilimumab	1
*RT series*	27
Prior RT-series	6
Normofractionationed RT-series	16
RT-dose	54 Gy (10.8 Gy-66.6 Gy)
RT fractionation	1.8 Gy (1.8 Gy-2 Gy)
Hypofractioned/Stereotactic RT-series	11
RT-dose	20 Gy (20 Gy-39 Gy)
RT fractionation	10 Gy (3 Gy-20 Gy)
*Toxicities*	
Anemia	12 (11 Grade 1, 1 Grade 2)
Thrombocytopenia	2 (Grade 1)
Lymphopenia	16 (8 Grade 1, 8 Grade 2)
Neutropenia	1 (Grade 3)
Mucositis	1 (Grade 3)
Erythema	8 (4 Grade 1, 2 Grade 2, 2 Grade 3)

n = 24 with 27 RT series

Gy, gray; RT, radiotherapy; y, year

After a median follow-up of 30.5 months, 6 patients revealed a local recurrence (mean local control: 44.3 m; 95% confidence interval (CI): 34.7–53.8 m) and overall PFS was 3 m (95% CI: 0–10.9 m).

**Fig. 1:**
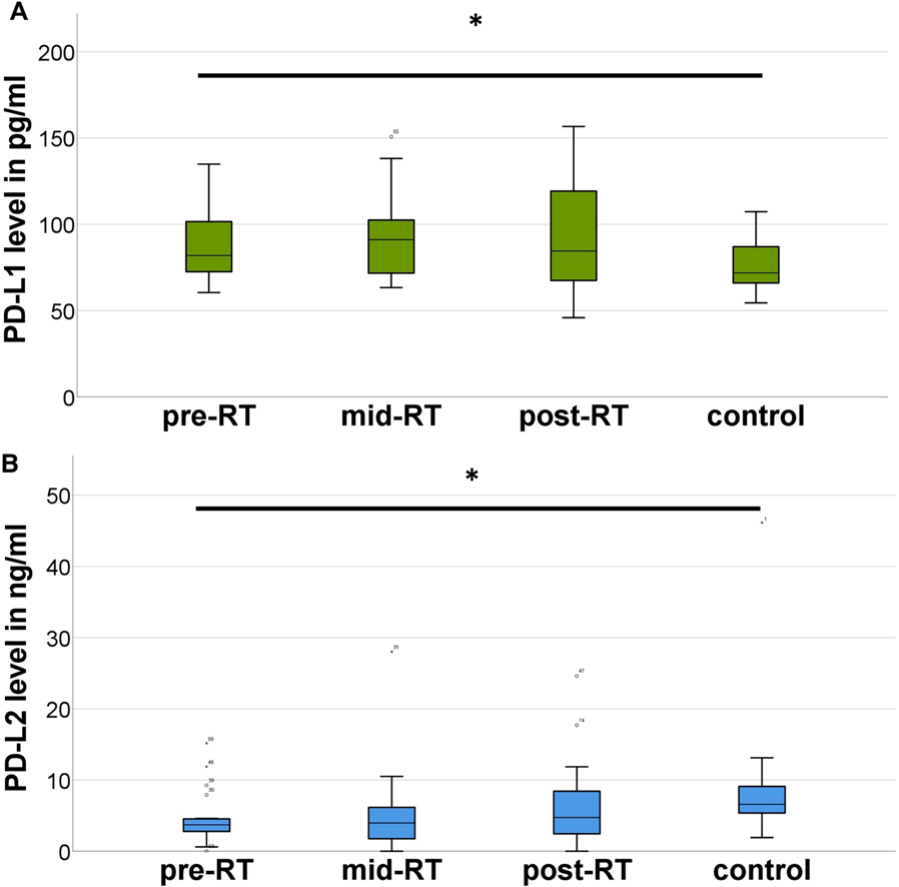
Box-plot-diagrams displaying the serum levels of PD-L1 (**A**) and PD-L2 (**B**) at different time points. Before (pre-RT), after completion of half (mid-RT) and after completion of the radiotherapy (post-RT). In comparison with healthy volunteers, a significant elevation of PD-L1 and decrease of PD-L2 was found (* = significance). RT, radiotherapy

There was a significant difference in the mean values of PD-L1 (90.1 pg/ml vs. 76.7 pg/ml for patients vs. control, respectively; *p* = 0.024; Table 2) and PD-L2 (4.4 ng/ml vs. 8.7 ng/ml; *p* = 0.04) between the baseline serum-level in the patients analyzed and the control collective, respectively (Fig. 1). Patients undergoing previous immunotherapy had a significantly higher PD-L1 level in comparison to patients without (104.0 pg/ml vs. 78.3 pg/ml; *p* = 0.003), which persisted during RT (mid-RT: 107.7 pg/ml vs. 80.2 pg/ml; *p* = 0.007; post-RT: 115.4 pg/ml vs. 75.3 pg/ml; *p* = 0.011). Increased PD-L1 values prior to therapy were not influenced by the type of immunotherapy (interferon vs. PD-L1 antibody *p* = 0.406). Patients treated with previous interferon uniformly revealed a decrease of PD-L1 in the mid-RT control (compared to pre-RT) which rose again for post-RT. No homogenous behavior for patients treated with PD-L1 antibodies was revealed. In contrast, no significant differences in PD-L1 levels pre-RT were found when patients with previous immunotherapy were excluded (patients: 78.3 pg/ml vs. control: 76.7 pg/ml; *p* = 0.772).

**Table 2:** Characterization of the immunological profile (n = 24)

	Patients	Control	*p*
PD-L1	90.1	76.7	0.024
PD-L2	4.4	8.7	0.04
IL-6	8.5	0.6	0.052
CTLA-4	153.7	267.2	0.295

Mean serum levels of inflammatory parameters compared between patients and healthy controls. PD-L1, IL-6 and CTLA-4 in pg/ml; PD-L2 in ng/ml. Significance was tested via two-sample t-tests

CTLA, cytotoxic t-lymphocyte-associated protein; IL, Interleukin; PD-L, programmed death-ligand

In a post-hoc power analysis the required sample sizes for significant results were estimated: for IL-6 (Cohens d = 0.564, $\alpha$ = 0.05) a power of 0.445 resulted in a minimum collective of 25 patients and 21 healthy controls, whereas CTLA-4 (Cohens d = 0.321, $\alpha$ = 0.05) required 72 patients and 60 healthy participants.

An elevation of PD-L1 or PD-L2 above the mean or median value did not herald a significant change in distant PFS or overall progression (data not shown).

Overall, 20 out of 24 patients underwent more than one blood draw and were eligible for dynamic investigation: comparisons were made between the blood analysis pre-RT, mid-RT and post-RT, revealing no significant dynamics (Table 3). This did not change when comparing normo- vs. hypofractionation treatments (data not shown). In contrast, when accounting for concomitant immunotherapy there was a significant difference between pre-RT and mid-RT for PD-L1 (84.8 pg/ml vs. 94.0 pg/ml for pre-RT vs. mid-RT; *p* = 0.044) in the group without immunotherapy.

**Table 3:** Dynamics of the inflammatory serum parameter during and after radiotherapy (RT) (n = 20)

	pre-RT	mid-RT	post-RT	*p* (pre vs. mid)	*p* (pre vs. post)
PD-L1	90.1	92.6	93.3	0.445	0.398
PD-L2	4.4	5.3	6.4	0.263	0.063
IL-6	8.5	8.4	9.5	0.913	0.920
CTLA-4	153.7	102.8	75.3	0.642	0.472

Mean serum levels of the respective parameters before (pre-RT), during (mid-RT) and after completion of RT (post-RT) were assessed. PD-L1, IL-6 and CTLA-4 in pg/ml; PD-L2 in ng/ml. Significance of mean values was tested via one-sample t-tests

CTLA, cytotoxic t-lymphocyte-associated protein; IL, Interleukin; PD-L, programmed death-ligand

Response assessment was possible for 20 RT series with 9 complete responses and 10 overall responses, both being not influenced significantly by PD-L1 or -2 levels above the mean (*p* = 0.642 or 1.0, respectively).

## Discussion

The hereby presented analysis outlines a distinctive immunological marker profile for melanoma patients with a decrease in soluble PD-L2 and increase in PD-L1, the latter being induced by previous immunotherapy. This work is one of the first to investigate the expression and dynamics of an immunological marker profile in the context of radiotherapy.

In comparison to healthy volunteers, patients (with melanoma) presented with significantly higher levels of sPD-L1 [12, 14], whereas other works did not describe this difference [13]. The increase may be triggered by previous immunotherapy (as in this work) or circulating inflammatory mediators: Interferon-y, as a pivotal inflammatory cytokine, has been identified at the interface between PD-L1 bearing tumor cells and tumor infiltrating leukocytes and is a potent inducer of sPD-L1 expression both in vitro and in vivo [12, 16].

Data on soluble PD-L2 are sparse and to our knowledge, no decisive analysis in melanoma patients has been conducted. In a population of patients with non-small cell lung cancer treated by nivolumab, sPD-L2 failed to predict response rate, PFS or OS, but was associated with grade 3 and 4 immune related adverse events [17]. In a larger analysis of lymphoma patients encompassing different entities, sPD-L2 was elevated and revealed distinctive levels between different types of diffuse large B-cell lymphoma [18]. In contrast, another work elaborated decreased levels of sPD-L2 in patients with epithelial ovarian cancer [19]. Importantly, the aforementioned investigations report values of sPD-L2 comparable to the numbers described here (1.86–18.25 ng/ml), corroborating validity of our data [17–19].

This analysis has some drawbacks, being mono-institutional and limited in patient number. As all melanoma patients were included, the collective reveals heterogeneity. Due to the anonymization process, the control group of healthy volunteers was not age-matched. Therefore, the results may be pre-liminary and the aim of the examination was rather hypothesis-generating than a proof-of-concept The set of biomarkers is limited and did not cover important regulators like interferon (s. above). In addition, the level of soluble biomarkers was low being in the range of pg/ml–ng/ml with a considerable variability between different patients (e.g. IL-6 ranged between 0.39 and 93.46 pg/ml). Consequently, Cordonnier and colleagues suggested to use circulating exosomal PD-L1 instead of the soluble version [20]. In the future, a larger and more homogenous patient collective will be attempted. In addition, as there is data illustrating the possible prognostic impact of biomarker changes in the long-term run (e.g. 5 months in [12]), further blood draws shall be attempted.

These implementations will likely shed more light on the dynamics and prognostic implementations of the soluble inflammatory mediators and immunological response in malignant melanoma.

## Data Availability

Not applicable.
